# Purification and biochemical characterization of two laccase isoenzymes isolated from *Trichoderma harzianum* S7113 and its application for bisphenol A degradation

**DOI:** 10.1186/s12934-022-02011-z

**Published:** 2023-01-02

**Authors:** Alshaimaa M. Elsayed, Mohamed Mahmoud, Ghada S. A. Abdel Karim, Mohamed Abdelraof, Abdelmageed M. Othman

**Affiliations:** 1 Molecular Biology Department, Biotechnology Research Institute, National Research Centre, Dokki, Giza, 12622 Egypt; 2 Water Pollution Research Department, National Research Centre, 33 El-Buhouth St., Dokki, Giza, 12622 Egypt; 3 Microbial Chemistry Department, Biotechnology Research Institute, National Research Centre, Dokki, Giza, 12622 Egypt

**Keywords:** Laccase, Purification, Biochemical characterization, Isoenzymes, Bisphenol A, Degradation

## Abstract

Two laccase isoenzymes (LacA and LacB) were isolated from a novel *Trichoderma harzianum* S7113 isolate employing ammonium sulfate precipitation, Sephadex G100, and DEAE Sepharose ion exchange chromatography. The molecular weights of the purified LacA and LacB laccases were estimated to be 63 and 48 kDa, respectively. The two isoenzymes had their optimum activities at the same temperature (50 °C), but at slightly different pH values (pH 3.0 for LacA and pH 2.5 for LacB). LacA and LacB had the same thermal stability at 40 °C and pH stability at pH 9.0. The two isoenzymes also showed a high level of specific activity toward ABTS, where the *K*_*m*_ values of LacA and LacB were 0.100 and 0.065 mM, whereas their *V*_*max*_ values were 0.603 and 0.182 µmol min^−1^, respectively. LacA and LacB catalytic activity was stimulated by Mg^2+^, Zn^2+^, K^+^, and Ni^2+^, whereas it was inhibited by Hg^2+^ and Pb^2+^, β-mercaptoethanol, EDTA, and SDS, and completely inhibited by sodium azide. Our findings indicate that purified laccase has a promising capacity for bisphenol A (BPA) bioremediation across a broad pH range. This finding opens up new opportunities for the commercialization of this technique in a variety of biotechnology-based applications, particularly for removing endocrine chemicals from the environment.

## Introduction

The blue multicopper oxidase enzyme laccase (EC 1.10.3.2) has the ability to catalyze the oxidation process of phenolic and non-phenolic aromatic substrates while also reducing molecular oxygen to water [1–3]. Using molecular oxygen as an electron acceptor, laccase oxidizes its substrates by an electron transfer mechanism that generates unstable free radical intermediates and causes non-enzymatic reactions that break down substrate molecules [4]. Laccase substrate specificity differs from one organism to another, and in the presence of adequate redox mediators, the range of laccase oxidizable substrates can be greatly extended [5]. Laccase is a glycosylated monomer with a molecular mass ranging from 54 to 97 kDa, relying on the source species. Numerous environmental pollutants, such as bisphenol A (BPA), may be successfully broken down and mineralized by fungi using their laccases [20, 21]. Laccase belongs to the polyphenol oxidases family, and its active site contains four copper atoms from types T_1_, T_2_, and T_3_ [6, 7]. The type-1 copper center is in charge of the substrate's initial oxidation, whereas the type-2 and type-3 copper types combine to form a trinuclear center, which is where the laccase catalytic activity occurs [4, 8, 9]. Type-3 copper sequesters the electrons that are transported from the substrate to Type-l copper and converts oxygen to water through a firmly linked peroxide intermediate, whereas Type-2 copper promotes the dissociation of the oxygen–oxygen link in the latter [22].

Laccases from all kingdoms have indeed been developed and described in hundreds of studies to date. Laccase is found in a wide range of higher plants, insects, bacteria, and mainly in fungi. *Rhus vernicifera*, a Japanese lacquer tree, is where laccase was initially discovered. Then laccase enzymes were subsequently discovered in a variety of plants, insects, and bacteria [10]. The ability of bacterial laccases to be generated both intracellularly and extracellularly, with active enzymes across a broad pH and temperature range, was investigated. For instance, laccase from *Bacillus subtilis* is most active at 75 °C, and at 80 °C, it has a 4 h half-life [11]. Following medium adjustment, laccase yield from *Pseudomonas aeruginosa* reached 46 U mL^−1^. Other instances of bacteria that generate laccases comprise *Streptomyces antibioticus, Pseudomonas putida*, *Campylobacter jejuni*, *E. coli*, *Bacillus* spp., and others [10]. The known fungal producers of laccase are deuteromycetes, ascomycetes, and basidiomycetes. Laccases from basidiomycetes and ascomycetes have been thoroughly examined; however, there are just a few studies on deuteromycetes laccase. Basidiomycetes, particularly white rot fungi, are thought to be effective laccase producers among them. Polyphenol oxidases have been found to be produced by several *Trichoderma* species, particularly *T. harzianum* [5, 12]. The majority of the laccases disclosed in the scientific literature have been confined and collected from almost all wood-decaying fungi, including *Trametes gallica*, *Pleurotus eryngii*, *Trametes villosa*, *Trametes hirsuta*, *Lentinus tigrinus*, *Trametes versicolor*, *Trametes ochracea*, *Coriolopsis polyzona*, and *Cerrena maxima* [13, 14].

Previous investigations on laccase biochemical properties declared that the majority of fungal laccases function best within 50 and 60 °C, as well as the *t*_*1/2*_ values of various laccases are temperature-dependent [2]. The ideal pH for laccases differs based on the substrate and its redox potential; in acidic environments (pH 3.0), laccases have had the largest impact on ABTS [9]. *Thermothelomyces thermophilus* [9], *Coriolus hirsutus* [8], and *Marasmius* sp. [15] laccases displayed their highest levels of activity when ABTS was used as a substrate at pH 3.0. Laccases originating from fungal origins are normally persistent at a little acidic pH, despite the fact that pH tolerance substantially differs relying on the enzyme source [2]. The laccases from *Colletotrichum lagenarium* [16], *Thielavia* sp. [17], and *Monilinia fructicola* [18] are persistent in the acidic pH (3.0–5.0), whereas the laccases from *Trametes* sp. [19] and *Agaricus bisporus* CU13 [13] are active in the alkaline pH (7.0–9.0). Various homologous laccases from *Chaetomium* sp. [20], *Shiraia* sp. [21], and *Elaeocarpus sylvestris* [22] all demonstrated a broad pH region (4.0–10.0). Laccase from *Bacillus* sp. was also very stable between pH 5.0 and 10.0, keeping over 80% of its reactivity for at least 24 h (pH 7.0–10.0) [23]. Low *K*_*m*_ values imply that laccase formulations have excellent affinity for their particular substrates [2, 9]. The *K*_*m*_ estimates of laccases from *Kabatiella bupleuri* G3 IBMiP (0.58 mM) [24], *Agaricus bisporus* CU13 Lacc1 and Lacc2 (0.394 and 0.158 μM) [25], *Colletotrichum lagenarium* (0.34 mM) [16], *Myceliophthora thermophila* (0.040 mM) [2], *Cryptococcus albidus* (0.8158 mM) [26], and *Thermothelomyces thermophilus* (0.051 mM) [9] were recorded with ABTS as a substrate.

Laccases play a role in plant disease, pigmentation, detoxification, and lignin degradation [27, 28]. These functions are associated with the oxidation of a wide range of organic compounds, such as monophenols, polyphenols, aromatic amines, and their derivatives [2, 5]. Many biotechnological processes involve laccases, such as bioremediation, the development of biosensors for the detection of polyphenols in wine and juice, organic synthesis, the bleaching of pulp in the paper industry, the decolorization of textile dyes, wastewater treatment, and the detoxification of pollutants, mainly due to their catalytic property and broad substrate specificity [4, 8, 9, 25, 29]. Also, the construction of oxygen reduction biocathodes in biofuel cells, biosensors, immunoassay labeling, and organic synthesis by biocatalysis are among the most widely investigated applications of laccases [8, 30].

In this context, laccases have been widely used for the efficient degradation of BPA, which is one of the most widely used phenolic chemicals for the production of polycarbonate and epoxy resins [31, 32]. More importantly, BPA has been identified as an endocrine-disrupting chemical, causing potential detrimental impacts on human health as well as wildlife [33, 34]. For example, BPA can cause metabolic disorders in children and breast cancer even with quite low concentrations in water bodies [35]. Although there are several proposed biotic techniques, such as ultrasonic oxidation, ozonation, photocatalytic oxidation, and advanced oxidation processes for BPA degradation; microbiological transformations, including enzymatic bioremediation, remain one of the most efficient and cost-effective approaches for achieving safe conversion of BPA [33, 36].

In the present study, the purification, biochemical characterization, kinetic constants, and stability properties of the laccase produced and optimized in our previous work [37] from a new potent laccase producer, *Trichoderma harzianum* S7113, were reported. The prospective uses of the purified laccase in the treatment of BPA were also mentioned.

## Materials and methods

### Chemicals

Guaiacol (2-methoxyphenol), 2,6-Dimethoxyphenol (DMP), Syringaldazine (4-hydroxy-3,5-dimethoxybenzaldehyde azine) (SGZ), Catechol (2-hydroxyphenol), Pyrogallol (2,3-dihydroxyphenol), ABTS (2,2ʹ-Azinobis-(3-ethylbenzothiazoline-6-sulfonic acid)), Bradford reagent for protein assay, Sephadex G-100, DEAE-Sepharose, Sodium dodecyl sulphate (SDS) and β-mercaptoethanol were purchased from Sigma-Aldrich Company (USA). Ammonium sulfate for protein precipitation was supplied by Merck (Germany). The study also employed other compounds, all of which were of analytical grade and didn't require any further purification.

### Enzyme source

The laccase enzyme produced and optimized, as mentioned in our previous work [37] from a new potent laccase producer, *T. harzianum* S7113, was used in the current study as the source of enzyme.

### Enzyme assay and protein estimation

In a 2.0-mL reaction mixture with 0.5 mL of 0.3 mM ABTS as substrate that is dissolved in sodium citrate buffer (0.1 M; pH 4.5), laccase activity was measured using an appropriate quantity of enzyme sample. Utilizing a Carry-100 Agilent UV–Vis Spectrophotometer (Germany), the change in absorbance was monitored for 1.0 min in order to detect the oxidation of ABTS at 420 nm (ε_420_ = 36 mM^−1^ cm^−1^). The quantity of enzyme necessary to oxidize 1 μmol of substrate per minute was used to define one unit of enzyme activity [9, 25, 37]. Using bovine serum albumin as the reference material, the Bradford [38] method was used to determine the protein content. All experiments were carried out in triplicate. Data is expressed using averages of results obtained.

### Purification of the laccase from *T. harzianum* S7113

Using ammonium sulfate fractionation at 4 °C and 40–80% saturation, the crude enzyme was precipitated. At 4 °C and 10500 ×*g* for 10 min, the mixture was centrifuged. The pellets were collected and dissolved in sodium phosphate buffer (0.1 M, pH 7.0), after which they were dialyzed for 24 h at 4 °C against sodium phosphate buffer (0.02 M, pH 7.0). After being dialyzed, the enzyme was added to a Sephadex G-100 column (2 × 80 cm) that had already been pre-equilibrated with sodium phosphate buffer (0.02 M, pH 7.0), and fractions of 4.0 mL were collected at a flow rate of 1.0 mL min^−1^. The laccase activity and protein of these fractions were then determined as described in Othman and Wollenberger [8]. A DEAE-Sepharose column (2 × 30 cm) had previously been equilibrated with Tris buffer (0.05 M, pH 8.0) when laccase-active fractions were mixed, concentrated, and added. Following a 100-mL wash with the same buffer, the bound proteins were gradually released from the column using NaCl gradients of varying concentrations (0.0–0.4 M) in the equilibration buffer. At a flow rate of 0.6 mL min^−1^, all chromatographic fractions were collected in 3.0 mL fractions, and the laccase activity and protein content were measured as before.

### SDS-PAGE analysis

To verify the enzyme's purity and determine the purified laccase's molecular weight, a sodium dodecyl sulfate polyacrylamide gel electrophoresis (SDS-PAGE) study was performed. According to Laemmli's technique [39], the SDS-PAGE was carried out using a 10% resolving gel and a 4% stacking gel utilizing BLUeye prestained protein ladder (Sigma-Aldrich, 94964).

### Impact of pH on laccase activity and stability

ABTS was used as a substrate in a buffer containing 0.1 M sodium citrate, with pH values ranging from 2.0 to 5.5, to determine the impact of pH optimum on laccase activity. By incubating the enzyme solution in 0.1 M citrate and Tris buffers (pH 3, 5, 7, and 9) at 40 °C for 2 h, the pH stability of the enzyme was examined. Utilizing ABTS as a substrate, the residual activity was calculated following incubation.

### The influence of temperature on the stability and activity of laccase

Variable temperatures between 30 and 90 °C were used to examine the impact of temperature on laccase activity. Prior to adding the enzyme and starting the reaction, the substrate (ABTS) and buffer (0.1 M sodium citrate, pH 4.5) were mixed and incubated at various temperatures for 5 min. The enzyme was incubated for three hours at different temperatures of 40, 50, 60, and 70 °C in a 0.05 M sodium phosphate buffer (pH 7.0) to determine its thermal stability. The remaining activity was then measured using ABTS as the substrate.

### Metal ions and inhibitors' effects on laccase activity

In a sodium phosphate buffer (0.05 M, pH 7.0) containing separate metal ions, including Mg^2+^, Cu^2+^, Zn^2+^, Ni^2+^, Pb^2+^, Al^3+^, K^+^, Na^+^, and Hg^2+^ at concentrations of 1.0 and 5.0 mM, the pure laccase was incubated for 10 min. The effect of inhibitors on laccase activity was determined by the pre-incubation of the enzyme with some inhibitors such as (EDTA), sodium azide (NaN_3_), sodium deodocyl sulphate (SDS) and β-mercaptoethanol at concentrations of 0.1 mM and 0.5 mM for 10 min. Then the substrate (ABTS) was added to start the reaction, and the remaining activity was measured.

### Kinetic parameters and substrate specificity

Utilizing several substrates at their appropriate pH levels, including ABTS, syringaldazine, 2, 6-dimethoxyphenol, guaiacol, catechol, and pyrogallol, the purified laccase's substrate specificity was examined. The activities of laccase towards different substrates were tested at a concentration of 5.0 mM, except ABTS, which was conducted at a concentration of 0.3 mM for 1.0 min at room temperature (28 °C ± 2). The rates of substrate oxidation were determined by measuring the rise in absorbance at the designated wavelengths, and the molar extinction coefficients were taken from the literature [40, 41]. The kinetic parameters (*V*_*max*_ and *K*_*m*_) of laccase-catalyzed oxidation of ABTS at different concentrations (0.025–0.4 mM) were calculated at pH 4.5. From the Lineweaver–Burk plots of the reciprocal of reaction velocities and substrate concentrations in the Michaelis–Menten equation, the kinetic constants were determined [42].

### Enzymatic degradation of BPA by the purified laccase

Batch experiments were conducted in the presence and absence of laccase to test the viability of employing pure laccase to break down BPA. Each experiment involved introducing a certain amount of a freshly isolated laccase solution to a 50 mL BPA solution with varying substrate concentrations (10–100 mg L^−1^) and beginning pH values (4.0–9.0) in order to assess the laccase's ability to break down BPA. We also optimized enzyme concentration by varying its concentration between 0.125 and 0.75 U mL^−1^ at a fixed substrate concentration (i.e., 20 mg L^−1^). To stop the enzymatic reaction at various time intervals, 3 mL of the reaction medium were taken out and promptly quenched with 1 mL of 0.5 M hydrochloric acid. The BPA content was then determined spectrophotometrically as per the APHA guidelines [43]. At room temperature (i.e., 25 ± 3 °C), all tests were performed in triplicate. The averages of the values acquired from the repeated runs are used to express the data in the figures.

### Statistical analysis

Unless otherwise stated, every experimental work was completed in triplicate. Both tables and figures display the mean values of the obtained results together with their standard deviation (SD) values.

## Results and discussion

### Purification of *T. harzianum* S7113 laccase

**Fig. 1:**
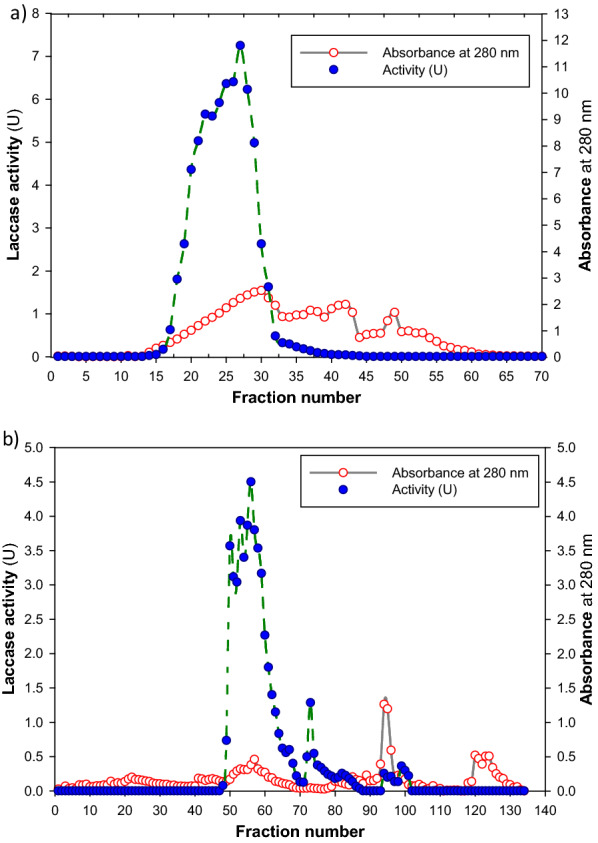
**a** Sephadex G-100 gel filtration chromatography; and **b** DEAE-Sepharose ion exchange profile of *T. harzianum* S7113 laccase isoenzymes

**Fig. 2:**
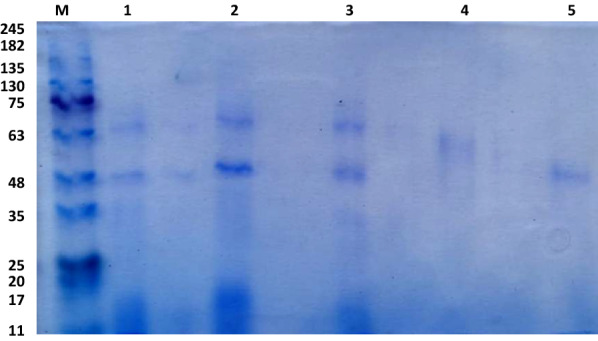
SDS-PAGE of *T. harzianum* S7113 laccase isoenzymes, M: marker, lane 1: crude laccase, lane 2: ammonium sulfate precipitation fraction, lane 3: Sephadex G-100 column fraction, lane 4: DEAE-Sepharose Lac A, and lane 5: DEAE-Sepharose Lac B

According to our earlier work [37], the laccase enzyme was extracted from *T. harzianum* S7113 in a crude form, and this crude extract was then purified utilizing a three-step process, which is outlined in Table 1. A purification fold of 2.73 and an increase in laccase specific activity to 2.98 U mg^−1^ protein were obtained in the first step of purification using ammonium sulfate (40–80%) precipitation (Table 1). The concentrated fraction from the previous step was subjected to gel filtration chromatography to get a purification fold of 5.34 (Table 1 and Fig. 1a). Then the concentrated enzyme was eluted via ion exchange chromatography (DEAE-Sepharose) to produce two laccase isoforms, Lac A (eluted by 0.1 M NaCl) and Lac B (eluted by 0.2 M NaCl), which have purification folds of 8.41 and 1.2, respectively (Table 1 and Fig. 1b). These results are close to those obtained from *Trametes polyzona* WRF03 laccase (purification fold of 13) using ammonium sulfate (90%), DEAE cellulose, and Sephadex G-100 columns [4] and from *Lentinus squarrosulus* MR13 yellow laccase (purification fold of 12.67) using ammonium sulfate (60%), DEAE cellulose, and Sephadex G-100 columns [27]. The purification fold for *Ganoderma lucidum* blue laccase, which was purified using DEAE-cellulose, Aff-gel blue gel, Con A-Sepharose, and FPLC-gel filtration on Superdex 75, was 25.4 (Wang and Ng, 2004). In contrast, the obtained fold is lower than that value. In this connection, laccase from *Trichoderma harzianum* strain HZN10 was purified to a purification fold of 25 using ammonium sulfate (70%), ultra-filtration, DEAE-Sepharose, and Sephadex G-100 chromatography [44]. The purified isoenzymes showed up on the SDS-PAGE profile as two separate protein bands. The molecular weights of the two isoenzymes were 63 and 48 kDa, respectively, as determined using SDS-PAGE (Fig. 2). Most fungal laccases were observed to have molecular weights between 50 and 90 kDa [6]. Laccases from *Pycnoporus sanguineus* (61.4 kDa) [40], *Lentinus squarrosulus *MR13 (66 kDa) [27], *Trametes polyzona *WRF03 (66 kDa) [4] and *H. echinacea* (63 kDa) (Wang and Ng, 2004) have molecular weights very close to that of Lac A. While laccases from *T. harzianum* strain HZN10 (56 kDa) [44] and *Alcaligenes faecalis* (50 kDa) [45] have a molecular weight close to that of Lac B. A lower molecular weight of 34 kDa is seen in *P. eryngii* laccase [46], whereas *Trametes versicolor* [47] and *T. harzianum* WL1 [5] laccases have larger molecular weights of 97 kDa and 79 kDa, respectively.

**Table 1:** Purification of laccase from *T. harzianum* S7113

Purification step	Total Protein (mg)	Total activity (U)	Specific activity (U mg^−1^ protein)	Recovery (%)	Purification fold
Crude	89.36	97.66	1.09	100	1.00
Ammonium sulphate ppt	22.34	66.66	2.98	68.25	2.73
Sephadex G-100	11.32	66.20	5.8	67.78	5.34
DEAE Sepharose					
0.1 M NaCl (Lac A)	4.46	41.06	9.19	42.04	8.41
0.2 M NaCl (Lac B)	3.66	4.88	1.3	5.08	1.22

### Temperature's impact on the stability and activity of laccases

**Fig. 3:**
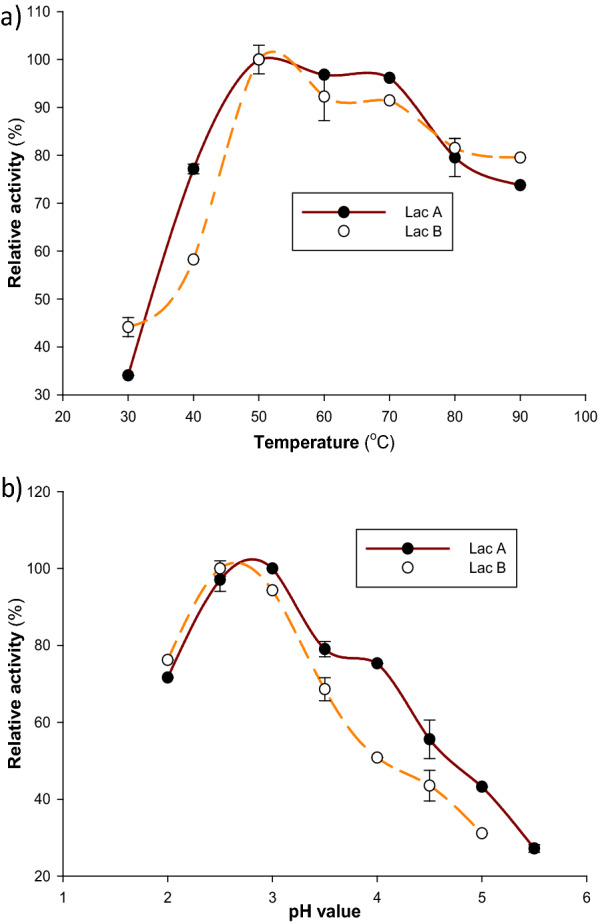
Effect of reaction **a** temperature and **b** pH on the activity of purified laccase isoenzymes. The buffer (0.1 M sodium citrate, pH 4.5) and substrate (ABTS) were combined and incubated at various temperatures for 5 min prior to adding the enzyme and beginning the reaction. To ascertain the effect of pH optimum on laccase activity, ABTS was utilized as a substrate in 0.1 M sodium citrate-containing buffers with pH ranges of 2.0 to 5.5

**Fig. 4:**
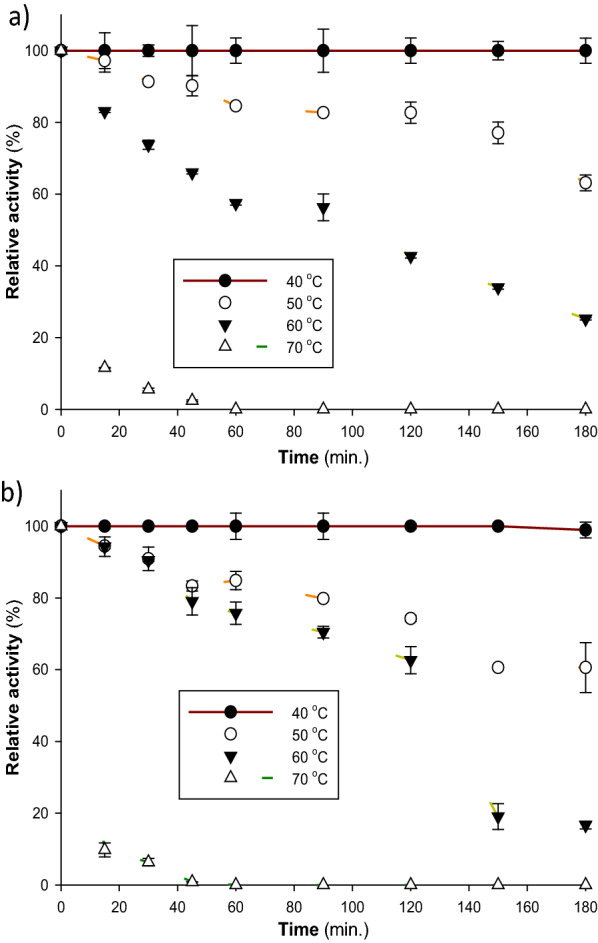
Effect of temperature on the stability of *T. harzianum* S7113 purified laccase isoenzymes **a** Lac A, and **b** Lac B. To ascertain the enzymes’ thermal stability, they were incubated for three hours at 40, 50, 60, and 70 °C in a 0.05 M sodium phosphate buffer (pH 7.0). Then, using ABTS as the substrate, the residual activity was quantified

The two *T. harzianum* S7113 laccase isoenzymes (Lac A and Lac B) showed optimum activity at a temperature of 50 °C, and the activity of the two isoenzymes started to decrease beyond 50 °C (Fig. 3a). These results are exactly similar to the temperature optima of laccases from *Pycnoporus sanguineus* [48], *Mycena purpureofusca* [49], and *Trametes versicolor* [47]. Moreover, *Agaricus bisporus* CU13 laccase (Lacc1 and Lacc2) isoenzymes [25], and *Trametes polyzona* WRF03 showed optimal activities at a temperature of 55 °C [4]. The obtained thermal stability results showed that Lac A and Lac B were completely stable at 40 °C for 3 h, as indicated in Fig. 4. Similarly, laccase from *Trametes polyzona* WRF03 was stable at temperatures range of 40 and 50 °C [4]. Additionally, laccases from *Pycnoporus sanguineus* [40] and *Pycnoporus cinnabarinus* [50] were stable up to 40 °C and below 50 °C. *Pycnoporus cinnabarinus* laccase was completely inactivated at 80 °C [50], which is in agreement with the current results. Laccases are generally stable between 30 and 50 °C but start to lose their activity beyond 60 °C [51].

### Impact of pH on laccase activity and stability

**Fig. 5:**
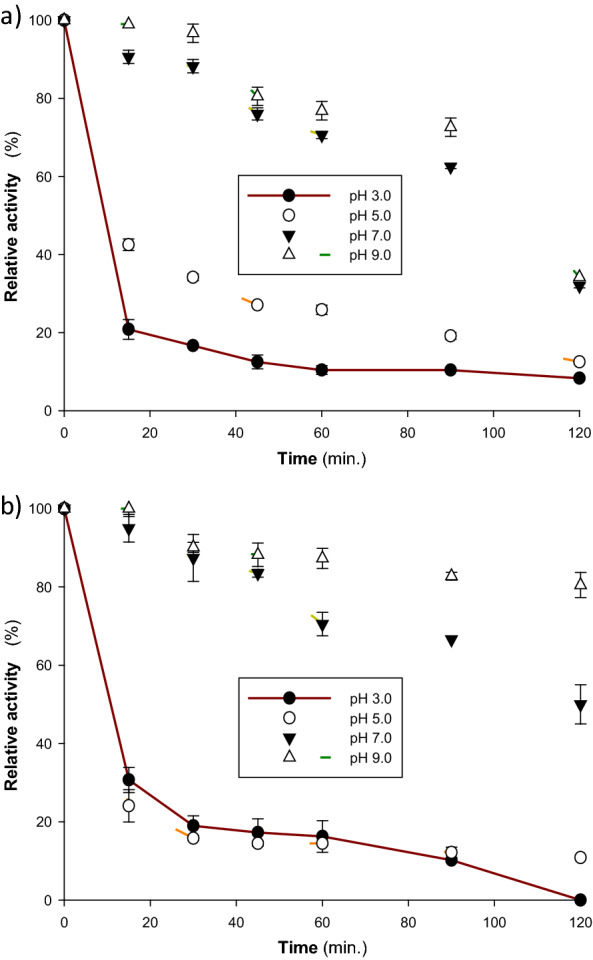
Effect of pH on the stability of *T. harzianum* S7113 purified laccase isoenzymes **a** Lac A, and **b** Lac B. The pH stability of the enzyme was investigated by incubating the enzyme solution in 0.1 M citrate and Tris buffers (pH 3, 5, 7, and 9) at 40 °C for 2 h. The residual activity was estimated after incubation using ABTS as the substrate

The two laccase isoenzymes (Lac A and Lac B) showed their maximum activities at pH 3.0 and 2.5, respectively (Fig. 3b). Numerous fungi laccases display pH optimum conditions in the acidic pH range, which might vary based on the source of the enzyme and the kind of substrate [8, 9, 52]. It ranges between pH 2.0 and 5.0 for ABTS, pH 3.0 and 8.0 for 2,6-dimethoxyphenol, and pH 3.5 and 7.0 for syringaldazine [6]. The optimum pH value of Lac A is similar to the optimum pH of laccase from *Pycnoporus sanguineus* [40] and laccase from *Trametes versicolor* (pH 3.0) using ABTS as a substrate [47], whereas the optimum pH of Lac B is close to that of laccase from *Trametes polyzona* WR710–1 (pH 2.2) [53]. At pH values higher than 5.0, the activity of both laccase isoenzymes toward ABTS was very low. The decrease in laccase activity at neutral or alkaline pH values may be caused by the hydroxyl anions' binding to laccase's T_2_/T_3_ copper center, which stops electrons from moving internally between T_1_ and T_2_/T_3_ tri-nuclear centers. This inhibits the enzyme's activity by preventing the binding of O_2_ as a terminal acceptor of electrons [8, 9]. Regarding the stability against pH, the two isoenzymes were more stable at neutral and alkaline regions than the acidic part. Whereas Lac A and Lac B had more or less the same pH stability at pH 7.0 for 2 h (Fig. 5), Lac B was more stable than Lac A at pH 9.0. These findings are resemble to the pH stability of laccase from *Perenniporia tephropora* at pH 8.0 [54] and laccase from *Cerrena unicolor* MTCC 5159 at pH 9.0 [55]. Othman et al. [25] reported that laccase isoforms from *Agaricus bisporus* CU13 were highly stable at pH 5.0 and 7.0 (Lacc1) and at pH 7.0 and 9.0 (Lacc2). The results of the pH stability of the *T. harzianum* S7113 laccase isoenzymes could be an extremely essential characteristic for their validity in industrial applications.

### Metal ions' effects on the activity of laccase isoenzymes

The two laccase isoenzymes from *T. harzianum* S7113 were tested for how metal ions affected their activity. As shown in Table 2, the activity of the two isoenzymes was increased gradually by increasing in some metal ions concentrations (from 1.0 to 5.0 mM) such as Mg^2+^, Zn^2+^, Ni^2+^, K^+^. Lac B activity was decreased by adding Na^+^ and more decreased by increasing Na^+^ concentration, whereas the activity of Lac A was increased by adding Na^+^ and further increase in its concentration has not any effect. The activity of both isoenzymes was inhibited by the addition of Cu^2+^, Pb^2+^, Al^3+^, and Hg^2+^ and further decreased by the increase in their concentrations (Table 2). The activity of laccase from *Marasmius sp.* BBKAV79 was completely inhibited by Hg^2+^ and decreased by Mg^2+^ at a concentration of 20 mM [1]. Additionally, the activity of laccase from *Pleurotus sp.* was completely inactivated by Zn^2+^ at a concentration of 2.0 mM [56]. At 1.0 mM Cu^2+^, *Lentinus squarrosulus* MR13 yellow laccase's activity increased, whereas it was slightly decreased by adding 1.0 mM of Ni^2+^, K^+^, Na^+^, Mg^2+^, and Hg^2+^ [27]. Moreover, the activity of laccase produced by *Mycena purpureofusca* was increased by adding 0.05 mM of Cu^2+^ and Zn^2+^; and was not affected by adding Mg^2+^ at the same concentration [49]. The activity of laccase from *Trametes polyzona* WRF03 was increased by adding 50 mM of Cu^2^ and Mg^2+^, whereas it was decreased by adding the same concentration of Zn^2+^ and Pb^2+^ [4]. The source of laccase and the kind of metal ions employed generally determine the effect of metal ions on laccase activity.

**Table 2:** Effect of metal ions on activity of *T. harzianum* S7113 purified laccase isoenzymes

Metal ions	Concentration (mM)	Relative activity (%)	
		Lac A	Lac B
Al^3+^	1.0	58.50 ± 0.22	52.16 ± 5.71
	5.0	37.73 ± 0.32	34.74 ± 0.52
Mg^2+^	1.0	122.7 ± 0.70	136.8 ± 5.88
	5.0	139.6 ± 2.30	157.9 ± 1.40
Zn^2+^	1.0	116.9 ± 1.72	105.3 ± 0.30
	5.0	143.4 ± 2.40	136.8 ± 1.72
K^+^	1.0	115.1 ± 2.18	110.5 ± 5.48
	5.0	130.2 ± 2.06	131.6 ± 5.60
Ni^2+^	1.0	116.9 ± 0.85	147.4 ± 1.76
	5.0	147.2 ± 1.21	173.4 ± 8.56
Hg^2+^	1.0	12.50 ± 0.71	35.30 ± 3.26
	5.0	9.4 ± 1.14	18.40 ± 1.62
Cu^2+^	1.0	90.60 ± 1.04	82.10 ± 2.55
	5.0	88.70 ± 0.99	47.40 ± 0.42
Na^+^	1.0	116.4 ± 1.77	86.80 ± 0.58
	5.0	116.9 ± 3.01	58.40 ± 5.19
Pb^2+^	1.0	47.20 ± 1.21	50.50 ± 3.81
	5.0	16.80 ± 0.30	11.60 ± 1.72

The pure laccase was incubated for 10 min in a sodium phosphate buffer (0.05 M, pH 7.0) with distinct metal ions. The reaction was then initiated with the addition of the substrate (ABTS), and the residual activity was assessed in comparison to the control with no metal ions

### Inhibitors' impact on laccase activity

The effect of inhibitors (sodium azide, EDTA, SDS, and β-mercaptoethanol) on the activity of the *T. harzianum* S7113 isoenzymes was recorded in Table 3. The activity of the two isoenzymes was decreased gradually by increasing the inhibitors concentrations from 0.1 to 0.5 mM. Additionally, sodium azide totally suppressed the activity of both isoenzymes by binding to the types 2 and 3 copper sites, which impairs internal electron transport and, in turn, inhibits the activity of the enzyme [5]. This is analogous to the relative inhibition effect caused by EDTA (10 and 25 mM) on the activity of *Pycnoporus sanguineus* laccase as a function of concentration increase. Furthermore, *P. sanguineus* laccase was strongly inhibited by increasing the NaN_3_ concentration from 0.1 to 1.0 mM [40]. In this connection, the activity of *Trametes polyzona* WRF03 laccase was completely inhibited by 10 mM of NaN_3_ and decreased by the same concentration of SDS and EDTA [4]. *Lentinus squarrosulus* MR13 yellow laccase’s activity was totally suppressed by 1.0 mM of NaN_3_, 1.0 and 5.0 mM of EDTA, 10 mM of SDS, and 100 mM of β-mercaptoethanol [27]. Similarly, the activity of the *Trichoderma harzianum* WL1 enzyme was completely inhibited by 20 µM of NaN_3_ and 25 mM of EDTA [5].

**Table 3:** Effect of Inhibitors on the activity of *T. harzianum* S7113 purified laccase isoenzymes

Inhibitors	Concentration (mM)	Relative activity (%)	
		Lac A	Lac B
NaN_3_	0.1	0.23 ± 0.02	0.69 ± 0.73
	0.5	0.00 ± 0.00	0.00 ± 0.00
EDTA	0.1	84.4 ± 2.62	97.7 ± 2.06
	0.5	56.6 ± 1.87	93.6 ± 1.66
SDS	0.1	55.5 ± 1.93	94.7 ± 1.65
	0.5	50.9 ± 2.45	63.9 ± 4.35
β-mercaptoethanol	0.1	52.1 ± 4.37	63.4 ± 3.01
	0.5	10.0 ± 2.47	7.90 ± 2.39

The enzyme was pre-incubated with different inhibitors for 10 min to ascertain the impact of inhibitors on laccase activity. Following the addition of the reaction's substrate (ABTS), the residual activity was assessed

### Substrate specificity

Laccases have the ability to delignify, decolorize, and detoxify dyes from effluents, remove stains from biomaterials, and remediate polluted environments because they have the potential to catalyze towards aromatic substrates (mostly phenols) [7, 57].Substrate specificity for both isoenzymes was determined against some laccase-specific substrates (Table 4). ABTS with a concentration of 0.3 mM showed the highest relative activity for both Lac A and Lac B and was the most suitable substrate for the two isoenzymes under study. The other studied substrates showed dissimilar specificities toward the two purified laccase isoforms in the following order: syringaldazine, 2,6-dimethoxyphenol, guaiaciol, pyrogallol, and catechol. Laccase from *Magnaporthe grisea* had the ability to oxidize the tested substrates in the order of: syringaldazine, L-3,4-dihydroxyphenylalanine (DOPA), ferulic acid, α-naphthol, hydroquinone, guaiacol, p-cresol, catechol, and 4-methylcatechol [58]. Moreover, laccase from *Trametes polyzona* WRF03 had the ability to oxidize these ordered substrates: ABTS, α-naphthol, o-dianisidine, pyrogallol, guaiacol, catechol, resorcinol, orcinol, and veratryl alcohol [7].

**Table 4:** Substrate specificity of purified *T. harzianum* S7113 laccase isoenzymes

Substrates	Concentration (mM)	Optimal pH	Molar extinction coefficient (ɛ) (M^−1^ cm^−1^)	Relative activity (%)	
				Lac A	Lac B
ABTS	0.3	3.0	36,000	0.36 ± 0.01	0.12 ± 0.001
DMP	5.0	5.5	49.6	0.13 ± 0.006	0.05 ± 0.007
SGZ	5.0	6.0	65.00	0.24 ± 0.009	0.12 ± 0.009
Guaiaciol	5.0	5.5	12.000	0.08 ± 0.013	0.02 ± 0.001
Catechol	5.0	5.0	2.211	0.03 ± 0.001	0.03 ± 0.007
Pyrogallol	5.0	7.0	4.40	0.04 ± 0.002	0.016 ± 0.00

At room temperature (28 °C ± 2), laccase's activity towards several substrates was examined for one minute at their respective pH levels. By monitoring the increase in absorbance at the chosen wavelengths, the rates of substrate oxidation were calculated

### Kinetic parameters of laccase

**Fig. 6:**
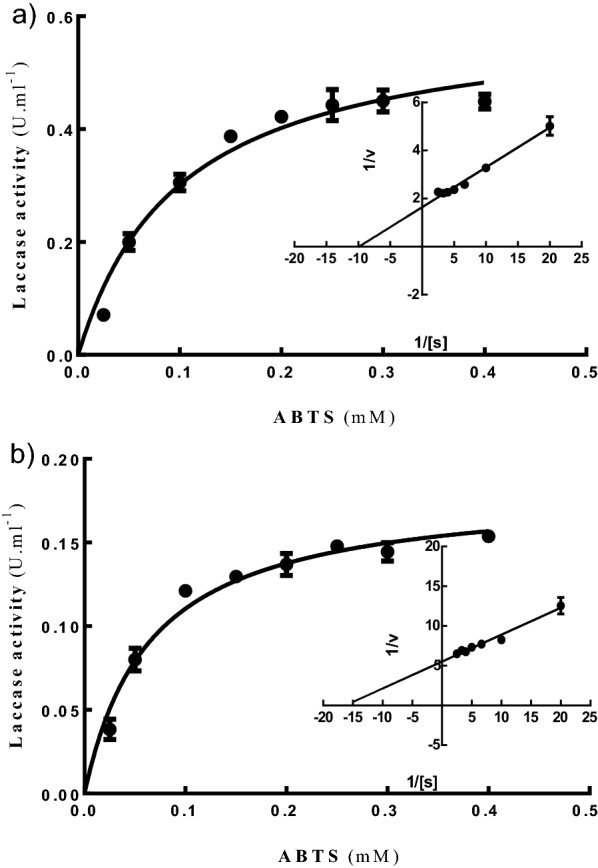
Kinetic profiles of **a** Lac A, and **b** Lac B purified isoenzymes*.* At pH 4.5, the kinetic parameters (*V*_*max*_ and *K*_*m*_) of the laccase-catalyzed oxidation of ABTS at various doses (0.025‒0.4 mM) were computed. The kinetic constants were derived using the Lineweaver–Burk plots of the reciprocal of reaction velocities and substrate concentrations in the Michaelis–Menten equation

The kinetic parameters (*K*_*m*_ and *V*_*max*_) are indications of the substrate specificity value, where the more a substrate is able to bind to an enzyme, the lower its *K*_*m*_ value, and the greater its substrate specificity [47]. Kinetic parameters were calculated for both isoenzymes using ABTS (Table 5 and Fig. 6). Both isoenzymes showed a high affinity for ABTS, similarly to other fungal laccases, but Lac B showed a lower *K*_*m*_ value (0.064 mM) for ABTS oxidation than Lac A (0.1 mM), which indicated that Lac B had a higher affinity for ABTS than Lac A. Moreover, the *V*_*max*_ value for Lac B was 0.182 μmol min^−1^, whereas the *V*_*max*_ value for Lac A was 0.603 μmol min^−1^. Similar findings were obtained using the *Lentinus squarrosulus* MR13 pure yellow laccase, which had *K*_*m*_ and *V*_*max*_ values of 0.0714 mM and 0.0091 mM min^−1^, respectively [27]. *Trametes sp.* AH28-2 laccase had *K*_*m*_ and *V*_*max*_ values of 0.025 mM and 0.67 mM min^−1^ mg^−1^ toward ABTS [59]. Additionally, using ABTS as the substrate, *Mycena purpureofusca* purified laccase had *K*_*m*_ and *V*_*max*_ values of 0.296 mM and 0.0645 mM min^−1^, respectively [49]. Purified laccase from *Trametes polyzona* WRF03 has *K*_*m*_ and *V*_*max*_ values of 0.00866 mM and 1.429 mM min^−1^, respectively, when employing ABTS as the substrate [4].

**Table 5:** *K*_*m*_ and *V*_*max*_ values of *T. harzianum* S7113 laccase isoenzymes

Enzyme	*V*_*max*_ (µmol min^−1^)	*K*_*m*_ (mΜ)
Lac A	0.603	0.100
Lac B	0.182	0.064

At pH 4.5, the kinetic parameters (*V*_*max*_ and *K*_*m*_) of the laccase-catalyzed oxidation of ABTS at various doses (0.025‒0.4 mM) were computed. The kinetic constants were derived using the Lineweaver–Burk plots of the reciprocal of reaction velocities and substrate concentrations in the Michaelis–Menten equation

### Biodegradation of BPA by fungal laccases

**Fig. 7:**
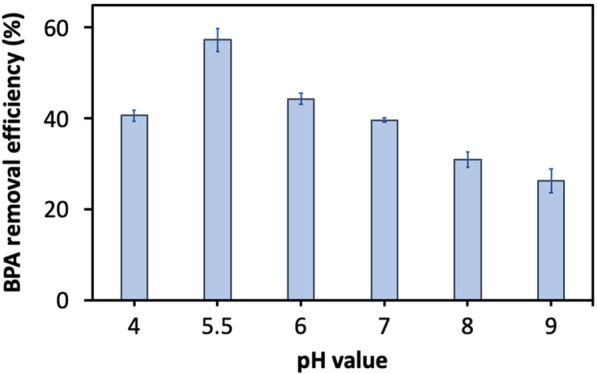
The dependence of the activity of the purified laccase at varied pH values. All experiments were carried out in triplicate at room temperature (25 ± 3 °C). The data in the figure is expressed using averages of the results obtained

Since the oxidative catalytic performance of laccase on a target substrate under a variety of pH and temperature conditions greatly influences its potential enzymatic activity, we assessed the catalytic potential of Lac A, considering that it is the major isoenzyme, for efficiently reducing BPA at various initial pH values in the range of pH 4‒9 with an initial BPA concentration of 20 mg L^−1^. We kept the initial laccase concentration at a relatively low level of 0.3 U mL^−1^ to only allow partial substrate utilization. Within the tested pH range, we observed that the BPA consumption reached its highest value at a pH value of 5.5 with over 57% BPA reduction (Fig. 7), which supports earlier research and demonstrates the ideal pH for producing a high level of enzymatic conversion of phenolic compounds is approximately pH 5–6 due to high laccase stability and strong metabolic interaction between laccase and substrate [37, 60]. Our results reveal a high bioremediation potential of the purified laccase over a wide pH range, implying an advantage for large-scale applications under varied conditions. Remarkably, the BPA concentration used in our study is much higher than BPA concentrations in different water and waste streams (e.g., industrial wastewater, rivers, and landfill leachates), which are in the range of 17.2–150 µg L^−1^.

**Fig. 8:**
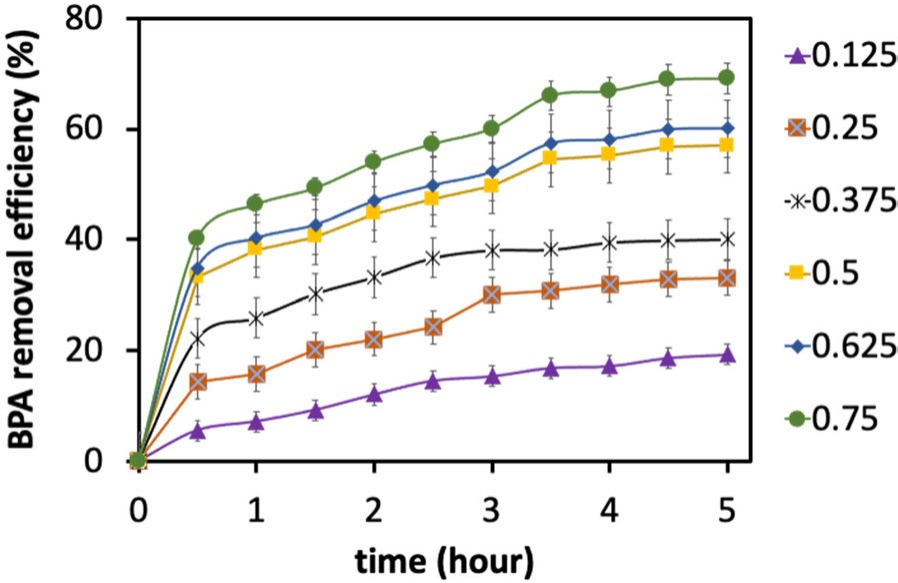
Effect of varied purified enzyme concentration (U/mL) on BPA removal efficiency. Conditions: BPA concentration = 20 mg L^−1^, pH = 5.5, and temperature = 25 °C. All experiments were carried out in triplicate. The data in the figure is expressed using averages of the results obtained

**Fig. 9:**
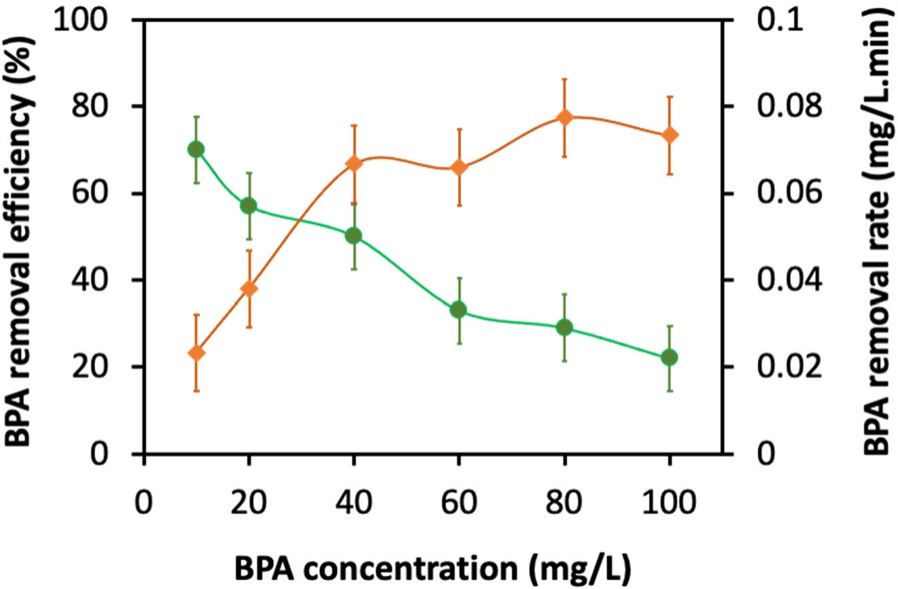
Effect of the initial BPA concentration on the BPA removal efficiency and rate within 5 h of treatment. Conditions: laccase concentration = 0.5 U mL^−1^, pH = 5.5, and temperature = 25 °C. All experiments were carried out in triplicate. The data in the figure is expressed using averages of the results obtained

We changed the initial enzyme concentration to assess how it affected the BPA conversion, while keeping the BPA concentration and initial pH constant at 20 mg L^−1^ and  pH 5.5, respectively, over the 5-h batch assays. We observed a gradual increase in BPA removal efficiency as the laccase concentration increased, peaking at 69% with a laccase concentration of 0.75 U mL^−1^ (Fig. 8). These results are consistent with previous studies that demonstrated enhanced BPA degradation by increasing the laccase enzyme concentration [61, 62]. Using a fixed enzyme concentration of 0.5 U mL^−1^ at a pH value of 5.5, the impact of the starting BPA concentration (in the range of 10–100 ppm) on the effectiveness of laccase's removal of BPA was also determined. Figure 9 shows that, up to a concentration of 60 mg L^−1^, the effectiveness of BPA removal dropped almost linearly; after that point, it became practically insensitive to the BPA concentration at the start of the experiment. A likely reason for this observation is that BPA removal was partially inhibited at high BPA concentrations due to the accumulation of free radicals produced during the enzymatic degradation of BPA, resulting in partial deactivation of laccase [63]. Our results are in agreement with a previous study, in which the removal efficiency of phenol was linearly decreased at high phenol concentrations in horseradish peroxidase-mediated reactions [64]. This catalytic capability of laccase to degrade BPA opens up new opportunities for the commercialization of this technique in different biotechnology-based applications, in particular for removing endocrine chemicals from the environment.

## Conclusions

The results of the current study indicate that the two isolated laccase isoenzymes (Lac A and Lac B) from the *T. harzianum* S7113 strain have excellent catalytic capabilities in terms of their pH optimum, pH stability, temperature optimum, thermal stability, and high affinity for various substrates. They can satisfy the requirements for a wide range of possible analytical, biomedical, and industrial applications owing to their characteristics. The catalytic capacity of laccase to break down BPA creates new prospects for the commercialization of this method in many biotechnology-based applications, particularly for eliminating endocrine chemicals from the environment.

## Data Availability

This article has all the data that was created or evaluated during this investigation.
